# Influence of Low-Magnitude High-Frequency Vibration on Bone Cells and Bone Regeneration

**DOI:** 10.3389/fbioe.2020.595139

**Published:** 2020-10-21

**Authors:** Lena Steppe, Astrid Liedert, Anita Ignatius, Melanie Haffner-Luntzer

**Affiliations:** Institute of Orthopedic Research and Biomechanics, Ulm University Medical Center, Ulm, Germany

**Keywords:** bone, fracture healing, vibration, LMHFV, osseointegration, mechanostimulation, regeneration

## Abstract

Bone is a mechanosensitive tissue for which mechanical stimuli are crucial in maintaining its structure and function. Bone cells react to their biomechanical environment by activating molecular signaling pathways, which regulate their proliferation, differentiation, and matrix production. Bone implants influence the mechanical conditions in the adjacent bone tissue. Optimizing their mechanical properties can support bone regeneration. Furthermore, external biomechanical stimulation can be applied to improve implant osseointegration and accelerate bone regeneration. One promising anabolic therapy is vertical whole-body low-magnitude high-frequency vibration (LMHFV). This form of vibration is currently extensively investigated to serve as an easy-to-apply, cost-effective, and efficient treatment for bone disorders and regeneration. This review aims to provide an overview of LMHFV effects on bone cells *in vitro* and on implant integration and bone fracture healing *in vivo*. In particular, we review the current knowledge on cellular signaling pathways which are influenced by LMHFV within bone tissue. Most of the *in vitro* experiments showed that LMHFV is able to enhance mesenchymal stem cell (MSC) and osteoblast proliferation. Furthermore, osteogenic differentiation of MSCs and osteoblasts was shown to be accelerated by LMHFV, whereas osteoclastogenic differentiation was inhibited. Furthermore, LMHFV increased bone regeneration during osteoporotic fracture healing and osseointegration of orthopedic implants. Important mechanosensitive pathways mediating the effects of LMHFV might be the Wnt/beta-catenin signaling pathway, the estrogen receptor (ER) signaling pathway, and cytoskeletal remodeling.

## Introduction

Bone is a mechanosensitive tissue which can react to changing loads by adapting bone mass and structure. Osteocytes are considered to be the main mechanosensor cell type in bone (Lanyon, 1993). However, other bone cells, including osteochondroprogenitor cells and osteoblasts, were also shown to react to their biomechanical environment by activating molecular signaling pathways, which regulate their proliferation, differentiation, and matrix production. Additionally, bone fracture healing critically depends on the mechanical conditions in the fracture area. Rigid fixation of long-bone fractures resulting in small interfragmentary movements induce direct intramembranous bone healing, whereas flexible fixation with higher interfragmentary movements results in callus healing with endochondral bone formation (Perren, 1979). Too flexible fixation can cause non-unions with hypertrophic fibrous tissue near the fracture gap. Similarly, too low biomechanical stimulation could be negative for bone healing. Regarding the underlying mechanism, it is proposed that mesenchymal cells are likely to form fibrous tissue under high stress conditions, whereas osseous tissue is generated under low stress conditions. At intermediate stresses, mesenchymal cells will differentiate into chondrocytes and initiate cartilaginous callus formation, which initially bridges the fracture gap (Claes et al., 2011). Therefore, the biomechanical environment appears to crucially influence bone cells during homeostasis, remodeling, and regeneration. Furthermore, the biomechanical environment plays a critical role during osseous implant integration. The implant material, its surface characteristics, and its biomechanical properties are major factors which should positively influence recruitment and differentiation of osteogenic cells to the implant surface to avoid implant loosening. Therefore, the development of new orthopedic implant materials and coatings is of great interest. Furthermore, because of the mechanosensitivity of bone, the application of external biophysical stimulation, including LMHFV, is considered to promote bone formation. This approach offers many benefits as a safe, easy-to-apply, and an effective treatment option which might be useful not only for preventing the risk of fractures but also for improving bone regeneration and implant osseointegration. Preclinical and clinical studies have already reported that vertical whole-body LMHFV is a successful anabolic strategy for healthy and osteoporotic patients to increase bone mineral density (Dubosc-Marchenay et al., 1992; Rubin et al., 2004; Ward et al., 2004; Xie et al., 2006; Wenger et al., 2010; Tezval et al., 2011). However, the underlying molecular pathways remain largely unknown. To shed some light on mechanotransduction pathways of LMHFV and the involved bone cells, *in vitro* studies with the focus on osteoblasts, osteoclasts, or their progenitors are reviewed. In addition, studies applying LMHFV during fracture healing or implant integration are discussed in this review article.

## Materials and Methods

The study was conducted in accordance with the PRISMA guidelines for systematic reviews. The electronic databases PubMed and Web of Science were reviewed regarding (1) influence of LMHFV on bone cells *in vitro* and the involved signaling pathways and (2) influence of LMHFV on bone regeneration *in vivo* (implant integration and fracture healing). The search strategies were focused on the PICOS criteria, which establish the inclusion and exclusion criteria as well as the combination of keywords for the studies to be analyzed. For search strategy (1), any controlled *in vitro* study, written in the English language was included. Studies including either mouse cell lines of osteoblasts (MC3T3-E1), osteoclasts (RAW264.7), or osteocytes (MLO-Y4) as well as MSCs or the respective primary cells derived from humans or rodents were considered. Only studies applying LMHFV (<1 *g*, *g*; 20–90 Hz) in a vertical manner were selected; therefore, acoustical and ultrasound oscillations were excluded. The primary outcome measures were proliferative capacities of the respective investigated cell type as well as their differentiation and changes in gene or protein expression. For (2), any preclinical study involving any animal species written in the English language was included. The primary outcome measures were callus formation, vascularization, bone formation and remodeling, mechanical stability of newly formed bone, and immune cell responses during fracture healing. Full papers of the final list of studies were reviewed and study data extracted and summarized in tables for subsequent analysis and discussion. In total, 51 articles were included in this paper. Vertical LMHFV was applied by either custom-made or commercially available vibration platforms in the studies reviewed in this article.

## Results

### Influence of LMHFV on Bone Cells *in vitro* and the Involved Signaling Pathways

#### MSCs

Mesenchymal stem cells are multipotent stem cells with the capacity to differentiate into different lineages, that is, osteogenic, chondrogenic, adipogenic, and myogenic (Pittenger et al., 1999; Kolf et al., 2007; Rastegar et al., 2010). The fate of MSCs is profoundly influenced by biomechanical stimuli (Pauwels, 1960). It is of great interest for the development of regenerative strategies to investigate whether LMHFV can favor MSC differentiation toward one specific lineage and which molecular mechanisms are involved.

Experimental *in vitro* studies used BMSCs or adipose-derived MSCs isolated from human, rat, or mouse BMSCs as well as the murine bone marrow stem cell line D1-ORL-UVA. To elucidate the effects of vibration, the cells were subjected to a specific vibration regime; however, loading parameters and time schedules varied among the studies (Table 1), but were within the range of 20–90 Hz and <1 *g* magnitude. It was demonstrated by the majority of studies, that LMHFV induced the expression of osteoblastic genes and osteogenic differentiation. In agreement with this finding, it was furthermore reported that LMHFV might alter lineage commitment of MSCs by inhibiting adipogenesis and promoting osteogenesis (Marycz et al., 2016; Baskan et al., 2017). This finding appears to be consistent regardless of the MSC origin or the vibration setting or duration. Because the culture conditions [two dimensional (2D) vs. three dimensional (3D)] are also known to influence MSC behavior and differentiation (Cukierman et al., 2002), some studies used 3D models (Zhou et al., 2011; Kim et al., 2012; Mehta, 2015; Chen et al., 2016), confirming the positive effect of LMHFV on osteogenic differentiation observed in 2D cultures. The study of Lau E. et al. (2011) reported inhibitory effects on osteogenesis, but this might be due to the relatively long vibration time of 1 h/day. Accumulating evidence also shows that LMHFV is able to increase MSC proliferation (Lau E. et al., 2011; Uzer et al., 2013; Demiray and Özçivici, 2015; Pongkitwitoon et al., 2016).

**TABLE 1:** LMHFV effects on mesenchymal stem cells.

References	Cells	Environment	Frequency (Hz)	Magnitude (*g)	Time schedule	Main outcome
Baskan et al., 2017	D1-ORL-UVA	TCP	90	0.15	15 min/day 7 days	Adipogenic markers ↓
Chen et al., 2016	rBMSCs	HA-coated titanium scaffolds (3D)	40	0.3	30 min/day 14 days	Expression of osteogenic markers (Runx2, Osx, Col-1, OC) ↑
Demiray and Özçivici, 2015	D1-ORL-UVA	TCP	90	0.15	15 min/day for 7 days	Cytoskeletal adaptations: total actin content and actin fiber thickness ↑ Cell proliferation ↑
Lau E. et al., 2011	rMSCs	TCP	60	0.3	1 h/day on days 1 + 2 and 4–6	Osteogenesis ↓
Li et al., 2019	rBMSCs	TCP (not stated)	45	0.9	30 min/day 5 days	Expression of osteogenic markers (Runx2, Osx, Col-1, OCN) ↑
Lu et al., 2018	BMSCs	TCP (not stated)	40	0.3	15 min/day	Cell proliferation and mineralization ↑ Expression of osteogenic markers (Runx2, Col-I, ALP, OPN, OC) ↑
Marycz et al., 2016	hASCs	TCP	25, 35, 45	0.3	15 min/day 14 days	Expression of osteogenic and chondrogenic markers (BMP-2, Col-II, Sox9) ↑ Adipogenesis ↓
Mehta, 2015	hMSCs	Synthetic 3D scaffold (PEGDA)	100	0.3 3 6	24 h	Osteogenic differentiation (ALP) and mineralization ↑
Kim et al., 2012	hMSCs	Collagen sponge (3D scaffold), TCP	30–40	0.3	10 min/day 5 days	Expression of osteogenic and vascularization-related markers (Col-I, OPG, VEGF, VEGF) ↑
Pongkitwitoon et al., 2016	hBMCs	TCP	30 or 100	0.15 1	2 × 20 min/day (2 h rest period)	Cell proliferation ↑ Expression of osteogenic markers (Runx2, ALP) ↑
Uzer et al., 2013	hASCs	TCP	30 or 100	0.15 1 2	30 min/day	Cell proliferation and mineralization ↑ Cytoskeletal remodeling ↑
Uzer et al., 2015	mBMSCs	TCP or collagen-I coated plates	90	0.7	2 × 20 min/day	Cytoskeletal remodeling ↑
Zhou et al., 2011	BMSCs	Human bone-derived scaffolds (3D)	40	0.3	30 min/12 h	Expression of osteogenic markers (Col-I, OCN, ALP, Runx2) ↑ ERK1/2 pathway involved

Cytoskeletal remodeling might be crucial for mechanotransduction (Helmke and Davies, 2002; Arnsdorf et al., 2009; Dahl et al., 2010). It was demonstrated by Uzer et al. (2013, 2015) that LMHFV upregulated actin-related genes and that a coupling between the nucleus and cytoskeleton is indispensable for amplifying the mechanoresponse and further promote cell signaling (Uzer et al., 2013, 2015).

#### Osteoblasts and LMHFV

Bone formation is mediated by osteoblasts, which are derived from MSCs through, among others, activation of the master transcription factor Runx2 (Cbfa1) (Komori et al., 1997). During osteoblast maturation, the cells undergo a differentiation process which is accompanied by extracellular matrix production and subsequent mineralization (Owen et al., 1990; Quarles et al., 1992; Lian and Stein, 1995). The terminal state of osteoblasts are osteocytes, which are long-living cells embedded into the bone matrix. Mechanical stimuli are crucial for osteogenesis and bone remodeling, while osteocytes (Lanyon, 1993) as well as osteoblasts (Neidlinger-Wilke et al., 1994) are considered to be mechanosensitive cells. Therefore, it appears likely that osteoblasts may be affected by LMHFV.

To assess the direct impact of vibration on osteoblasts, several studies were conducted using primary mouse osteoblasts or osteoblast-like cell lines (MC3T3-E1, SaOS-2) (Table 2). While applying vibration settings ranging over 30–60 Hz at 0.25–0.5 *g*, cell proliferation, mineralization, cytoskeletal remodeling, and gene expression were evaluated. Gao et al. and Haffner-Luntzer et al. reported a significantly increased osteoblast proliferation in contrast to the study from Apa et al. showing decreased proliferation. This inconsistency might be due to the use of SasOS-2 cells (Apa et al., 2018) in comparison to primary osteoblasts (Gao et al., 2017) or MC3T3-E1 cells (Haffner-Luntzer et al., 2018b), because SaOS-2 cells are already more differentiated. Quantitative Alizarin Red-S staining showed that also extracellular matrix mineralization was increased by LMHFV (Gao et al., 2017). Furthermore, gene and protein expression levels of osteogenesis-related mediators and pathways were evaluated. It was demonstrated that mechanical vibration significantly increased mRNA expression of ALP, OCN, Runx2, BMP, Osterix, type I collagen, and OPG, whereas SOST was downregulated (Ota et al., 2016; Gao et al., 2017). All these markers are important for bone formation and osteoblast differentiation, except SOST, which has the opposite effect. Therefore, LMHFV appears to accelerate osteogenic differentiation of osteoblasts. Moreover, the vibration treatment significantly increased gene expression levels of the Wnt signaling pathway members Wnt3a, Lrp6, and β−catenin (Gao et al., 2017), which are important for osteoblastogenesis. Using human iliac crest-derived mandible osteoblasts, Pravitharangul et al. reported that the RANKL/OPG mRNA ratio was reduced by LMHFV, suggesting an anti-resorptive cell response. Notably, another study by García-López et al. demonstrated decreased RANKL and increased IL-4, IL-13, IL-17, OPG, and TGF-β levels. Using the conditioned medium from osteoblasts on osteoclasts, the authors demonstrated that osteoclasts were inhibited. These results confirm previous findings that LMHFV promotes osteogenesis-associated gene expression and reduces osteoclastic mediators.

**TABLE 2:** LMHFV effects on osteoblasts.

References	Cells	Frequency (Hz)	Magnitude (*g)	Time schedule	Main outcome
García-López et al., 2020	Primary BALB/c mouse calvarial osteoblasts	30	0.25	20 min/day	IL-4, IL-13, IL-17, OPG, TGF-β1 ↑ RANKL ↓
Pravitharangul et al., 2018	Osteoblast−like cells from iliac crest and mandibular bone specimens	0, 30, 60	0.49	30 min/day	IL−6 mRNA expression ↑ IL−1β, RANKL mRNA ↓ RANKL/OPG ratio ↓ in iliac osteoblasts
Haffner-Luntzer et al., 2018b	MC3T3-E1 cells and primary C57BL/6 mouse osteoblasts	45	0.3	20 min/day	Cytoskeletal remodeling: actin content, actin fiber thickness ↑ Cell metabolic activity, cell proliferation ↑ ERα signaling involved
Apa et al., 2018	Osteoblast like cells (SaOS-2)	30, 60, 90	0.3 1 3	1 h/day	Proliferation ↓ (0.3 *g*, 30 Hz)
Gao et al., 2017	Primary osteoblasts	45	0.5	1 h/day, 3 days	ALP, OCN, Runx2, BMP, OPG ↑ SOST ↓ Proliferation ↑ Matrix mineralization ↑ Cytoskeletal remodeling Wnt signaling involved
Ota et al., 2016	MC3T3-E1	30, 60, 90	1.0–10 m/s^2^		Runx2, Osterix, Col-1, ALP ↑
Rosenberg et al., 2002	Samples of cancellous bone collected from femoral necks	20, 30, 60	1.0–10 m/s^2^		ALP ↑ (30–60 Hz)

Considering that external biophysical stimulation has been implicated in regulating actin cytoskeletal remodeling in MSCs, this was also examined in osteoblasts by Haffner-Luntzer et al. (2018b), showing that actin remodeling is increased by LMHFV treatment. In addition, Gao et al. demonstrated an increased number of microfilaments and thicker stress fibers in the vibrated group, suggesting that the vibration-induced effects on osteoblasts might be dependent on cytoskeletal rearrangement. Moreover, mechanotransduction in bone tissue is considered to be critically dependent on the presence of estrogen and its receptors (ERs) (Frost, 1987). Therefore, it was hypothesized that particularly ERα might be crucial for the effects of LMFHV on osteoblasts. Haffner-Luntzer et al. investigated the effects of 45 Hz (0.3 *g*) vibrations on MC3T3-E1 cells in estrogen-free medium and demonstrated higher metabolic cell activity and increased Ptgs2 gene expression (Cox2) after LMHFV treatment, whereas these findings were reversed in the presence of estrogen. Cox2 is known to be upregulated by mechanical strain and might be involved in osteoblast proliferation. To evaluate the role of ERα in mechanotransduction, siRNA knockdown was performed to block ERα signaling. In the absence of estrogen, it was demonstrated that ERα is indeed required for the increased Ptgs2 gene expression and proliferation of MC3T3-E1 cells after LMHFV suggesting that ligand-independent activity of ERα is responsible for the vibration-induced effects. The same effect was observed by adding the selective ERα antagonist MPP dihydrochloride. Therefore, estrogen and the ER pathway appear to play an important role in LMHFV-mediated mechanotransduction.

In conclusion, LMHFV regulates osteoblast proliferation, differentiation, and matrix mineralization via upregulation of Wnt-related gene expression, cytoskeletal remodeling, and ER pathways. Furthermore, it appears to enhance the expression of osteoclast-inhibiting factors.

#### Osteoclasts and LMHFV

In addition to its indirect effects on osteoclastogenesis via upregulation of anti-osteoclastic factors in osteoblasts, LMHFV might also directly act on osteoclasts (Table 3). These cells derive from the monocyte/macrophage hematopoietic lineage and are essential for bone resorption. Osteoclast differentiation and activity are induced by RANKL binding; however, this can be attenuated by OPG, a soluble RANKL-binding decoy receptor (Simonet et al., 1997; Khosla, 2001). Therefore, RANKL is frequently used as a cell culture supplement to induce osteoclast differentiation in RAW246.7 cells *in vitro* (Hsu et al., 1999).

**TABLE 3:** LMHFV effects on osteoclasts.

References	Cells	Frequency (Hz)	Magnitude (*g)	Time schedule	Main outcome
Sakamoto et al., 2019	RAW264.7	48.3	0.5	1 min	Proliferation ↑ No effect on osteoclast differentiation
Wu et al., 2012	RAW264.7	45	0.3	15 min/day	Inhibited actin ring formation MMP-9, cathepsin K, TRAP mRNA ↓ RANKL-induced osteoclast differentiation ↓

To determine whether LMHFV affects osteoclast functions, preosteoclastic murine RAW246.7 cells were subjected to LMHFV at 45 Hz (0.3 *g*) for 15 min/day (Wu et al., 2012) and subsequently analyzed for TRAP-positive multinucleated cells (MNCs) and osteoclast-specific gene expression. Notably, LMHFV significantly reduced the formation of TRAP-positive MNCs and the upregulation of the osteoclastic genes cathepsin K, MMP-9, and TRAP. While TRAP and cathepsin K are essential for bone resorption, MMP-9 mediates the migration of precursor cells toward the bone. Furthermore, the number of formed actin rings, which represents an important osteoclast adhesion structure, was reduced by LMHFV (Wu et al., 2012). By contrast, Sakamoto et al. (2019) reported a significant increase in pre-osteoclastic RAW246.7 cell proliferation 48 h after vibration for 1 min at 48.3 Hz (0.5 *g*). However, evaluation of TRAP-positive MNCs showed that osteoclast differentiation was not altered, which might be due to the fact that the cells only received one very short vibration treatment for 1 min. In co-culture experiments with RAW246.7 and previously vibrated osteocyte-like MLO-Y4 cells, the authors showed that osteoclast differentiation was significantly higher, which might be because of a significantly increased RANKL/OPG ratio in the supernatant of vibrated MLO-Y4 cells.

Although there are only three studies investigating the effects of LMHFV on osteoclasts, it appears that osteoclasts are rather inhibited *in vitro.* However, further studies investigating the involved underlying mechanism are needed.

#### Osteocytes and LMHFV

Osteocytes are derived from osteoblasts and are embedded into the mineralized bone matrix. They orchestrate bone remodeling by regulating osteoblast and osteoclast activity. Additionally, they secrete factors to stimulate or inhibit bone formation or resorption and they communicate with surrounding cells via dendritic processes through the lacuna-canalicular system. Because osteocytes are considered as the main physiological mechanosensor in bone tissue, they might also be affected by LMHFV (Table 4).

**TABLE 4:** LMHFV effects on osteocytes.

References	Cells	Frequency (Hz)	Magnitude (*g)	Time schedule	Main outcome
Thompson et al., 2015	Stem cell derived-osteocytes (SCD-O)	90	0.7	2 × 20 min/day (> 3 h in between) for 3 days	SOST↓ No changes in osteocyte differentiation or mineralization, as well as RANKL or OPG expression
Uzer et al., 2014	MLO-Y4 cells	30,100	0.15 1	30 min/day	Gap junctional intracellular communication (GJIC) ↑ Akt-signaling involved
Sakamoto et al., 2019	MLO-Y4 cells	48.3	0.5	1 min	RANKL mRNA ↑ NF-κB activation ↑

To investigate the effects of LMHFV, Thompson et al. applied LMHFV (90 Hz, 0.7 *g*) to stem cell-derived osteocytes originating from BMSCs and found no changes in the osteocytic marker genes Dmp1, Fgf23, or E11, suggesting that LMHFV has no effect on osteocytic differentiation or mineralization. However, exposure to LMHFV significantly reduced SOST mRNA expression, but did not affect RANKL or OPG. Because SOST is a well-known inhibitor of osteoblastic bone formation, this suggests that LMHFV led to reduced osteocytes SOSTt expression and, therefore, increased osteoblastic bone formation.

By contrast, Sakamoto et al. reported enhanced RANKL mRNA expression by vibration of MLO-Y4 cells, whereas OPG levels were unaffected. This indicates an indirect positive effect of LMHFV on osteoclast differentiation via osteocytes. On the molecular level, GJIC through connexin 43 might be involved in vibration-induced cell mechanotransduction (Ziambaras et al., 1998; Cheng et al., 2001; Cherian et al., 2003; Batra et al., 2012). While Uzer et al. (2014) demonstrated that LMHFV (0.15 *g*, 30 Hz, 30 min/day) significantly increased GJIC between MLO-Y4 cells by 25%, this effect was, however, dependent on Akt activation.

In conclusion, LMHFV appears to activate RANKL expression and to reduce SOST in osteocytes, whereas osteocytic differentiation marker genes are not directly affected.

### Influence of LMHFV on Bone Regeneration

Bone regeneration after fracture requires a complex interplay between a variety of different cell types and biological mediators. In addition, an appropriate mechanical stimulation at the fracture site is considered to be essential for the healing process. Therefore, introducing external biomechanical stimuli by LMHFV is a promising strategy that might provoke positive effects on bone formation. In the following chapters, the influence of LMHFV on the early, intermediate, and late phases of fracture healing as well as on implant osseointegration is reviewed.

#### Influence of LMHFV During the Early Inflammatory Phase After Fracture

Within the early inflammatory phase, a complex interaction of several cell types and molecular mediators is essential for subsequent callus development and successful fracture repair. Danger- and pathogen-associated molecular patterns derived from the hematoma recruit neutrophils to the fracture site, which in turn release various mediators leading to the migration of macrophages, lymphocytes, and other immune cells. Macrophages together with neutrophils remove cell debris and secrete cytokines and chemokines to recruit progenitor cells and promote bone regeneration. The local inflammatory response is among others regulated by the pro-inflammatory mediators TNF-α, IL-1, and IL-6 and the anti-inflammatory IL-10 (Claes et al., 2012; Kovtun et al., 2016).

Only a few studies have investigated the effects of LMHFV on inflammation. Chow et al. investigated whether LMHFV influences the early inflammatory response after fracture in osteoporotic rats, because in estrogen-deficient, osteoporotic animal models, the innate immune response after fracture was shown to be altered (Chow et al., 2019). However, there is no clear consensus on whether the inflammation is lower (Chow et al., 2019) or rather increased (Haffner-Luntzer et al., 2017; Fischer et al., 2018). Chow et al. (2019) found that LMHFV induced a phenotype switch from pro-inflammatory M1 to pro-regenerative M2 macrophages. M2 macrophages were previously demonstrated to promote osteogenic differentiation of progenitor cells (Zhang et al., 2017); therefore, this could represent a mechanism by which LMHFV might influence the fracture healing outcome. The effect of vibration on other immune cell types and mediators that are involved in early inflammation has to date not been investigated.

The formation of new blood vessels (neo-angiogenesis) in the soft callus is important for bone repair. A decreased blood supply at the fracture site is associated with a compromised healing outcome in animal models and patients (Miclau et al., 2017; Haffner-Luntzer et al., 2019). Cheung et al. (2012) examined the effect of LMHFV on angiogenesis during fracture healing in rats and could demonstrate by 3D high-frequency power Doppler and microangiography that the vascular volume, blood flow, and angiogenesis were significantly higher in the LMHFV-treated rats compared to the sham-treated animals. In spite of the fact that neo-angiogenesis in OVX rats is compromised, this was attenuated by vibration in OVX rats. Because this is the only report on vascularization and LMHFV during fracture healing, more research is needed to verify the results and to gain further knowledge about the molecular mechanisms.

#### Influence of LMHFV on Cartilaginous and Bony Callus Formation

During endochondral fracture healing, a cartilaginous callus is formed at the fracture site to initially bridge the gap and provide some stability. During callus maturation, chondrocytes become hypertrophic, the cartilaginous matrix starts to calcify, and bone formation is initiated to replace the soft callus with bone. Because callus formation and maturation after fracture is highly relevant for successful healing and is significantly influenced by the biomechanical environment at the fracture site, applying mechanical stimulation by LMHFV could be beneficial for promoting fracture healing. Furthermore, because it is well known that OVX rodents and osteoporotic patients (Nikolaou et al., 2009) exhibit a significantly impaired callus maturation (Beil et al., 2010), many studies investigating the effects of LMHFV on fracture healing were performed with both OVX and sham-OVX rats or mice.

The majority of rat studies used a femur osteotomy model with internal fixation combined with a 35 Hz and 0.3 *g* vibration treatment (Table 5). Strikingly, all rat studies demonstrated that LMHFV application accelerates physiological fracture healing and it has been shown that LMHFV is further able to rescue OVX-induced impaired healing (Shi et al., 2010; Chung et al., 2014). LMHFV appears to promote callus formation by increasing both callus width and area between 1 and 3 weeks after fracture (Shi et al., 2010; Chow et al., 2011, 2016; Cheung et al., 2012). Histomorphometric analyses suggested that callus mineralization and maturation is also accelerated by LMHFV (Leung et al., 2009; Chung et al., 2014) because the amount osseous tissue was significantly higher while significantly less cartilage was formed in the vibration groups independently of the estrogen status of the animals (Chung et al., 2014). By contrast, a study by Shi et al. (2010) reported no changes in cartilage formation in OVX or sham-OVX rats on vibration treatment, which agrees with the finding of Leung et al. (2009) that a larger cartilage formation was only observed within the first week of vibration treatment, but not at later time points (2 or 4 weeks) in estrogen-competent rats.

**TABLE 5:** LMHFV effects on fracture healing using rat models.

References	Strain	Frequency (Hz)	Magnitude (*g)	Time schedule	Groups	Main outcome
Cheung et al., 2012	SD rats	35	0.3	20 min/day, 5 days/week 2/4/8 weeks	OVX Sham	Bone formation at 2/4 weeks ↑ Angiogenesis ↑
Chow et al., 2011	SD rats	35	0.3	20 min/day, 5 days/week 2/4/8 weeks	OVX	Bridging rate ↑ Callus remodeling ↑ mineralization ↑ Reversed the effects of ibandronate
Chow et al., 2016	SD rats	35	0.3	20 min/day, 5 days/week 2/4/8 weeks	OVX Sham	Bony callus formation ↑ ER expression ↑ in OVX
Chow et al., 2019	SD rats	35	0.3	20 min/day, 5 days/week 1/2/4/8 weeks	OVX Sham	Mechanical stability ↑ Cox2-upregulation in callus augmented by NSAID usage Promoted switch of macrophage polarization from M1 (pro-inflammatory) to M2 (anti-inflammatory)
Choy et al., 2020	SD rats	35	0.3	20 min/day, 5 days/week 1/2/6 weeks	OVX Sham	Lacuna-canalicular network outgrowth ↑ Mineralization ↑ Both effects stronger in OVX
Chung et al., 2014	SD rats	35	0.3	20 min/day, 5 days/week 2/4/8 weeks	OVX Sham	Chondrogenesis-, osteogenesis- and remodeling-related genes ↑ (Col-2, Col-1, RANKL/OPG) Cartilaginous tissue area ↓ in OVX
Gao et al., 2016	Female rats (strain not stated)	35	0.25	(1) 15 min/day; (2) 3 × 5 min (>4 h apart); (3) 7 days 15 min/day, then 7 days rest (4) 7 days 3 × 5 min (>4 h apart), then 7 days rest 4 weeks	Non-OVX	Bone formation and mechanical stability ↑
Leung et al., 2009	SD rats	35	0.3	20 min/day, 5 days/week 1/2/4 weeks	Non-OVX	Bone formation and mechanical stability ↑
Shi et al., 2010	SD rats	35	0.3	20 min/day, 5 days/week 2/4/8 weeks	OVX Sham	Bone formation and mechanical stability ↑ Callus formation ↑ mineralization ↑ Non-OVX bones were less sensitive to mechanical stimulation

In mice, LMHFV improved fracture healing only in OVX animals (Table 6), while in estrogen-competent mice, delayed healing was observed (Wehrle et al., 2014). In detail, Wehrle et al. demonstrated that the flexural rigidity and bone formation in the fracture callus were significantly decreased in estrogen-competent mice that received a vibration treatment of 45 Hz at 0.3 *g* acceleration. Interestingly, the application of 35 Hz had no effect on the fracture callus, highlighting that the vibration frequency is a crucial parameter that should be carefully considered. By contrast, LMHFV with 45 Hz and 0.3 *g* significantly increased flexural rigidity and bone formation in the fracture callus in estrogen-deficient, OVX mice (Wehrle et al., 2015). Therefore, estrogen appears to play a crucial role in mediating the effects of LMHFV on bone healing. On a molecular level, the expression of ERs appears to play an important role in the context of accelerated fracture healing induced by LMHFV. It was shown that ERα expression was enhanced by LMHFV only in the fracture callus of OVX mice (Wehrle et al., 2015) and OVX rats (Chow et al., 2016), both, on the mRNA and protein levels. This might explain the higher mechanical sensitivity of osteoporotic bone toward LMHFV, because ERα is suggested to have a mechanosensory function. By contrast, ERβ expression was upregulated by LMHFV in sham-OVX mice. A study performed by Haffner-Luntzer et al. (2018a) used both global ERα and ERβ knockout mice with a femur diaphysis osteotomy model and a vibration regime of 45 Hz and 0.3 *g* to evaluate the role of ERs in vibration-induced effects on bone regeneration. Biomechanical testing and micro-computed tomography analysis revealed that ERα is required for the effects of LMHFV on fracture healing both in sham-OVX and OVX mice, whereas ERβ was shown to play a minor role.

**TABLE 6:** LMHFV effects on fracture healing using mouse models.

References	Strain	Frequency (Hz)	Magnitude (*g)	Time schedule	Groups	Main outcome
Haffner-Luntzer et al., 2018a	ERα-KO, ERβ-KO (C57BL/6)	45	0.3	20 min/day, 5 days/week 3 weeks	OVX Sham	Flexural rigidity ↑ ERα required for beneficial effect of LMHFV on healing in OVX mice
Wehrle et al., 2014	C57BL/6 (12 weeks)	35, 45	0.3	20 min/day, 5 days/week 10 days or 3 weeks	Non-OVX	35 Hz: no effect 45 Hz: flexural rigidity ↓ bone formation in the fracture callus ↓
Wehrle et al., 2015	C57BL/6 (49 weeks)	45	0.3	20 min/day, 5 days/week 10 days or 3 weeks	OVX Sham	Flexural rigidity ↑ in OVX Bone formation↑ in OVX
Zhang et al., 2020	SAMP8, non-sarcopenic SAMR1	35	0.3	20 min/day, 5 days/week 2/4/6 weeks	Non-OVX	Callus formation ↑ Callus remodeling ↑ Mechanical properties ↑ in non-sarcopenic mice

In large animals like sheep, several studies confirmed that LMHFV can improve fracture healing in a metatarsal osteotomy model (Table 7). Li et al. (2018) and Mu et al. (2019) compared the efficacy of intermittent and continuous vibration and showed that LMHFV had the greatest effect in the 7-day interval group (71.4% recovery with grade 3 healing vs. 42.9% in the continuous treatment group and 14.3% in the natural healing group without vibration. Callus volumes were also significantly increased in the 7-day interval group (Li et al., 2018), confirming the results in rodents. By contrast, Tan et al. (2017) found that the vibration treatment with 1-day intermittence had the greatest positive effect on fracture healing. This discrepancy could be explained by the 1-week earlier vibration initiation in the study by Tan et al. and the slightly different vibration settings which might lead to differences in the healing outcome. On a molecular level, increased serum levels of bone remodeling markers (BALP, BGP, TRAP) as well as of endochondral ossification markers (TGF β1) were found in all vibration groups.

**TABLE 7:** LMHFV effects on fracture healing using sheep models.

References	Species	Frequency (Hz)	Magnitude (*g)	Time schedule	Main outcome
Li et al., 2018	Small-tail Han sheep	35	0.25	15 min/day continuous or intermittent at 1, 3, 5, 7, 14 days Start: 14 days post-OP	Callus formation ↑ mechanical properties ↑ Ca, P, Ca/P ratio ↑
Mu et al., 2019	Short-tailed Han sheep	35	0.25	15 min/day continuous or intermittent at 1, 2, 3, 5, 7, 17 days Start: 14 days post-OP	Callus formation ↑
Tan et al., 2016	Short-tailed Han sheep	35	0.25	15 min/day or intermittent at 7 days Start: 14 days post-OP	Callus volume ↑ Bone elastic modulus ↑ Ca, P, Ca/P ratio ↑
Tan et al., 2017	Small-tail sheep	35	0.3	20 min/day intermittent at 1, 3, 5 or 7 days Start: 7 days post-OP	Callus width and area ↑ week 4: ALP, BGP, TGFβ1 ↑ TRAP5b ↓ week 8: TGFβ1 ↑ TRAP5b ↓

In conclusion, the majority of studies both in small and large animal models demonstrated that LMHFV is able to accelerate fracture healing by increasing bone formation in the fracture callus. In mice, the effects of LMHFV appear to be highly dependent on the estrogen status of the animals. In this model organism, LMHFV provoked negative effects on fracture healing in estrogen-competent animals, whereas it improved healing in estrogen-deficient, osteoporotic animals. Further research is needed to investigate the underlying molecular mechanisms.

#### Influence of LMHFV on Callus Remodeling

Following bony bridging of the fracture gap, the external callus is continually remodeled by osteoclasts until the normal bone structure and shape are restored. Because osteoclasts were shown to be target cells of LMHFV *in vitro* (Wu et al., 2012; Sakamoto et al., 2019), vibration treatment might influence callus remodeling during fracture healing.

Shi et al. (2010) and Chung et al. (2014) showed that LMHFV induced more rapid callus remodeling particularly in OVX rats by upregulation of the RANKL/OPG ratio. The same authors investigated whether LMHFV influences callus remodeling in OVX rats by combining the vibration treatment with ibandronate administration, a bisphosphonate which inhibits osteoclast activity (Chow et al., 2011). As expected, bone remodeling was reduced in the animals treated with ibandronate alone. Notably, the combination of the anti-resorptive therapy with LMHFV ameliorated the effects of the bisphosphonate on callus remodeling. However, the authors did not evaluate osteoclast numbers or activity directly; therefore, drawing a valid conclusion about the effects of LMHFV on osteoclasts *in vivo* was not possible. By contrast, Haffner-Luntzer et al. did evaluate osteoclast numbers, but could not observe any effect of LMHFV both under estrogen-deficient or estrogen-competent conditions.

#### Influence of LMHFV on Osseointegration of Bone Implants

Bone implant osseointegration is a critical step toward preventing implant failure during bone regeneration. The implant material and its surface characteristics are major factors which might positively influence recruitment and differentiation of osteogenic cells to the implant surface to avoid implant loosening. Furthermore, the mechanical conditions at the bone–implant interface crucially influence bone regeneration and osseointegration (Albrektsson and Jacobsson, 1987; Hudieb et al., 2011). External biophysical stimulation by LMHFV might be an option to optimize osseointegration, because its anabolic potential for bone has already been demonstrated in numerous studies.

To examine the effect of LMHFV during implant osseointegration, rat models with titanium implants and a vibration regime of 0.2–0.5 *g* and 30–45 Hz have been used (Table 8). In response to LMHFV, all studies observed a significant increase in bone formation around the used implants (Ogawa et al., 2011a, b, 2014; Jing et al., 2015, 2018; Ruppert et al., 2018), even with the slightly different loading protocols that were applied. Bone volume to tissue volume ratio around the implant was analyzed by μCT scanning, whereas histomorphometrical analysis analyzed bone formation directly by fluorescence labeling of the newly build bone. Beyond that, LMHFV application significantly increased the bone mineral density in the bone-implant interface in the group that did receive LMHFV treatment (Liang et al., 2014), as analyzed by μCT scanning. These findings were further strengthened by biomechanical testing, which demonstrated significantly increased removal torque in the vibration group compared to the control animals (Liang et al., 2014).

**TABLE 8:** LMHFV effects on osseointegration of implants.

References	Strain	Frequency (Hz)	Magnitude (*g)	Time schedule	Implant	Study outcome
Chen et al., 2012	SD rats	30–35	0.3	20 min/day, and 5 days/week 8 weeks	Hydroxyapatite (HA)−coated titanium implants (proximal tibia)	BIC, BF ↑ but effects are weaker than alendronate Max. push out force ↑
Jing et al., 2015	Female New Zealand rabbits	30	0.5	1 h/day for 6 or 12 weeks	Porous titanium alloy (Ti6Al4V) (femoral condyle)	Bone ingrowth within pores of the implant ↑ BV/TV ↑ Tb. N ↑ ALP, OCN, Runx2, BMP2, OPG ↑ SOST, RANKL ↓
Liang et al., 2014	SD rats	45	0.2	30 min/day	Titanium implant (metaphyseal tibia)	BF, BIC ↑ thickness of the bone lamellae (TBL) ↑ BMD ↑ removal torque ↑
Ogawa et al., 2011b	Wistar rats	15 consecutive frequency steps (12, 20, 30, …, 150 Hz)	0.3	11 min/day 5 days/week 3, 7, 14, 25 days	Custom−made titanium implant (proximal metaphysis)	BIC, BF ↑
Ogawa et al., 2011a	Male Wistar rats	15 consecutive frequency steps (12, 20, 30, …, 150 Hz)	0.3	1.25, 2.5, 5 and 2 × 1.25 min (>4 h)	Custom-made titanium implant (medio-proximal site of tibia)	BIC ↑ 2 × 1.25 min most pronounced effect
Ogawa et al., 2014	Male Wistar rats	12–30/70–90/130–150	0.3/0.075/0.043	1/4 weeks	Titanium implant (metaphyseal tibia)	BIC ↑ BV/TV ↑
Ruppert et al., 2018	SD rats	55	0, 0.15, 0.3, 0.6, or 1.2	6 weeks	Titanium implant (tibia)	BF ↑
Shibamoto et al., 2018	Wistar rats	50	0.5	15 min/day 5 days/week	Titanium implant (metaphyseal tibia)	BIC ↑ LMHF + PTH had additive effects in OVX rats removal torque ↑
Zhou et al., 2015	SD rats	40	0.3	30 min/12 h 5 days/week for 12 weeks	Hydroxyapatite-coated titanium implants (medio-proximal site of tibia)	BV/TV, Tb.N, Tb. Th ↑ Runx2, OPN, OC ↑ RANKL ↓

Further studies investigated whether LMHFV is also capable of improving osseointegration in osteoporotic animals (Chen et al., 2012; Liang et al., 2014; Shibamoto et al., 2018). Indeed, LMHFV rescued OVX-induced compromised implant osseointegration by increasing the bone contact to the implant, the amount of newly formed bone and the shear strength at the interface (Chen et al., 2012; Liang et al., 2014; Shibamoto et al., 2018). This was demonstrated by histomorphometrical analysis.

Chen et al. treated OVX rats with LMHFV or by bisphosphonate alendronate administration and demonstrated that both treatments significantly increased osseointegration; however, the effects of LMHFV were less compared to alendronate. Hypothesizing that LMHFV and anti-osteoporosis medications might have additive effects on implant osseointegration, Shibamoto et al. compared the effects of PTH or alendronate treatment in OVX rats with or without LMHFV vibration. Only PTH and LMHFV displayed positive additive effects on implant integration (Chen et al., 2012).

Jing et al. (2015) analyzed the expression of osteogenic markers genes in rabbits which were subjected to LMHFV for 6 or 12 weeks. The osteogenesis-related genes Alpl, Bglap, Runx2, Bmp2, and Opg were upregulated, suggesting that LMHFV promotes osteoblastogenesis and mineralization. The osteoanabolic canonical Wnt pathway also appears to be activated, because Wnt3a, Lrp6, and β-catenin were expressed significantly higher in the LMHFV group, whereas anti-osteogenic SOST expression was significantly reduced (Chen et al., 2016). Moreover, the mRNA levels of osteoclastogenesis-associated Rankl were significantly reduced. Furthermore, ERK1/2 signaling, which is known to inhibit osteoclast activity, was shown to be upregulated after LMHFV (Zhou et al., 2015). In conclusion, LMHFV appears to activate osteogenic and to inhibit osteoclastogenic pathways. Nonetheless, further studies are needed to fully elucidate the mechanisms of vibration-induced bone formation around implants.

In summary, LMHFV improved osseointegration of titanium implants in estrogen-competent and deficient animal models by promoting osteoblastogenesis and exhibiting anti-resorptive effects.

## Conclusion

Mechanical stimuli are considered to be essential regulators in bone remodeling and regeneration. Therefore, external biophysical stimulation with LMHFV could possibly be used to enhance bone formation. It is supposed not to cause any side effects and can be readily applied by whole-body vibration.

Indeed, the first clinical studies demonstrated improved bone mineral density and bone mass after LMHFV in both healthy and osteoporotic patients. Underlying mechanisms have been extensively studied *in vitro* and *in vivo*. Most of the *in vitro* experiments showed that LMHFV is able to enhance MSC and osteoblast proliferation. Furthermore, osteogenic differentiation of MSCs and osteoblasts was shown to be accelerated by LMHFV. One important mechanosensitive pathway mediating the effects of LMHFV might be the Wnt/beta-catenin signaling pathway. Furthermore, it was demonstrated that ER signaling plays a crucial role particularly on osteoblasts. Ligand-dependent ER signaling might rather act negatively on osteogenic cell proliferation, whereas ligand-independent ER signaling mediated positive LMHFV effects. Additionally, cytoskeletal remodeling was induced by LMHFV, which might influence MSCs cell fate decision, proliferation, and differentiation. In addition to the effects of LMHFV on osteogenic cells, vibration treatment appears to inhibit osteoclast formation and activity directly and indirectly by reduced osteoclastogenic mediator release from osteoblasts.

During fracture healing, LMHFV may also exert diverse effects on the involved cell types. Vibration was shown to modulate macrophage polarization and enhance vascularization. Furthermore, LMHFV increased bone formation in the fracture callus. However, some studies demonstrated that this was only the case in estrogen-deficient, osteoporotic animals and that ER signaling was crucial for those effects. Therefore, attention has to be paid when transferring LMHFV to the clinical situation, because only specific fracture patient cohorts might benefit from whole-body vibration. Further research is needed to understand the involved molecular mechanisms, side effects, and potential benefits for patients. Before vibration can be recommended as a new treatment option for fractures, more clinical studies are needed to examine the efficacy, regimes, and safety for fracture healing. To date, there are no related clinical studies published in PubMed; however, two studies on that topic are registered at clinicaltrials.gov. In addition to its effects on fracture healing, LMHFV demonstrated positive effects on the osseointegration of orthopedic implants by increasing BIC and BF. Moreover, in this context, osteoanabolic Wnt/beta-catenin signaling was shown to mediate positive effects of LMHFV on bone formation. Furthermore, diminished osteoclastogenesis by reduced RANKL expression of osteoblasts was demonstrated.

In conclusion, LMHFV might be a promising treatment strategy to improve bone regeneration during fracture healing and implant integration. However, more research is needed to elucidate the involved molecular signaling pathways, since not much is known how LMHFV transduce mechanical stimulation into biochemical signals (Table 9). In particular, the ER signaling pathway was demonstrated to play a double-faced role by mediating negative effects of LMHFV on osteoblasts in the presence of estrogen, but positive effects in its absence. This might account for differences in fracture healing outcome after LMHFV in estrogen-competent and estrogen-deficient mice. Clinical trials are needed to investigate the translational potential of LMHFV and to define fracture patient cohorts which might benefit from this treatment. Indeed, some clinical trials showed a beneficial outcome of LMHFV on bone parameters (Rubin et al., 2004; Ward et al., 2004), whereas others demonstrated no effect of the vibration (Slatkovska et al., 2010; Lau R. et al., 2011). This might be due to different patient cohorts, but also due to different vibration regimes and ways to apply the biomechanical stimulation demonstrating how important it is to apply LMHFV in a very precise and controllable manner.

**TABLE 9:** LMHFV and involved molecular signaling pathways.

References	Pathways
Chow et al., 2016, 2019	Estrogen receptor (ER) Cox-2/prostaglandin signaling
Demiray and Özçivici, 2015	Cytoskeletal remodeling
Gao et al., 2017	Wnt signaling Cytoskeletal remodeling
Haffner-Luntzer et al., 2018a, b	ERα Cytoskeletal remodeling
Li et al., 2019	ERα Activation of the canonical Wnt pathway
Sakamoto et al., 2019	NF-κB
Uzer et al., 2013, 2014, 2015	Cytoskeletal remodeling Akt signaling
Zhou et al., 2011	ERK1/2 signaling

## Author Contributions

LS conducted the systematic literature research. LS and MH-L drafted the manuscript. All authors worked on the final version of the manuscript.

## Conflict of Interest

The authors declare that the research was conducted in the absence of any commercial or financial relationships that could be construed as a potential conflict of interest.

## Abbreviations

Akt: protein kinase B
ALP: alkaline phosphatase
BF: peri-implant bone formation
BIC: bone-to-implant contact
BMP: bone morphogenetic protein
BMSCs: bone marrow-derived MSCs
Col-1: collagen type I
Cox2: cyclooxygenase 2
ER: estrogen receptor
ERK1/2: extracellular signal-regulated kinase 1/2
*g*: gravitational acceleration
GJIC: gap junctional intracellular communication
Hz: hertz
IL: interleukin
LMHFV: low-magnitude high-frequency vibration
Lrp6: low-density lipoprotein receptor-related protein 5
MMP: matrix metallopeptidase
MSCs: mesenchymal stem cells
OCN: osteocalcin
OPG: osteoprotegerin
OPN: osteopontin
OVX: ovariectomy
PTH: parathyroid hormone
RANKL: receptor activator of nuclear factor-kappa B ligand
siRNA: small interfering RNA
SOST: sclerostin
TGF-β: transforming growth factor beta
TNF-α: tumor necrosis factor alpha
TRAP: tartrate resistant acid phosphatase.
